# A Flexible and Stretchable Triboelectric Nanogenerator with Agarose/P(HEA‐co‐AA)‐Al/NaCl Electrodes for Bio‐Mechanical Energy Harvesting and Fall Detection

**DOI:** 10.1002/open.202400394

**Published:** 2025-03-05

**Authors:** Xiwei Liu, Hui Zhang

**Affiliations:** ^1^ Sports College Lanzhou City University Lanzhou City Gansu Province 730070 China; ^2^ Military Sports Department Changchun Sci-Tech University Changchun 130600 China

**Keywords:** conductive hydrogel, fall detection, self-powered sensing, Triboelectric nanogenerators (TENGs)

## Abstract

Recent advancements in wearable electronics for fall detection have shown significant potential, yet challenges remain in developing reliable, energy‐efficient systems capable of continuous monitoring in real‐world conditions. In this work, a double‐network (DN) conductive hydrogel, comprising NaCl‐coordinated agarose and poly (2‐hydroxyethyl acrylate‐co‐acrylic acid) (Agarose/P(HEA‐co‐AA)‐Al/NaCl) (APA‐hydrogel), was synthesized. The agarose forms a stable first network, while the second network is established by P(HEA‐co‐AA) through aluminum ion coordination. Immersion in NaCl solution leads to the formation of hydrated sodium ions ([Na(H_2_O)n]^+^), which are anchored within the hydrogel matrix via hydrogen bonding and metal coordination. The resulting APA‐hydrogel was applied as a triboelectric nanogenerator (APA‐TENG), demonstrating excellent performance with an open‐circuit voltage of 900 V, a short‐circuit current of 73.42 μA, and a peak power output of 3.52 mW at a 3 MΩ load. APA‐TENG shows strong potential for energy harvesting and powering low‐power devices, as well as real‐time sensing for motion and fall detection, making it highly suitable for wearable and assistive technologies powered by human activity.

## Graphical abstract

A double‐network (DN) conductive hydrogel (APA‐hydrogel) was synthesized using NaCl‐coordinated agarose and poly (2‐hydroxyethyl acrylate‐co‐acrylic acid). Applied as a triboelectric nanogenerator (APA‐TENG), it achieved 900 V open‐circuit voltage, 73.42 μA short‐circuit current, and 3.52 mW peak power. APA‐TENG holds promise for energy harvesting, low‐power devices, and real‐time motion and fall detection in wearable technologies.

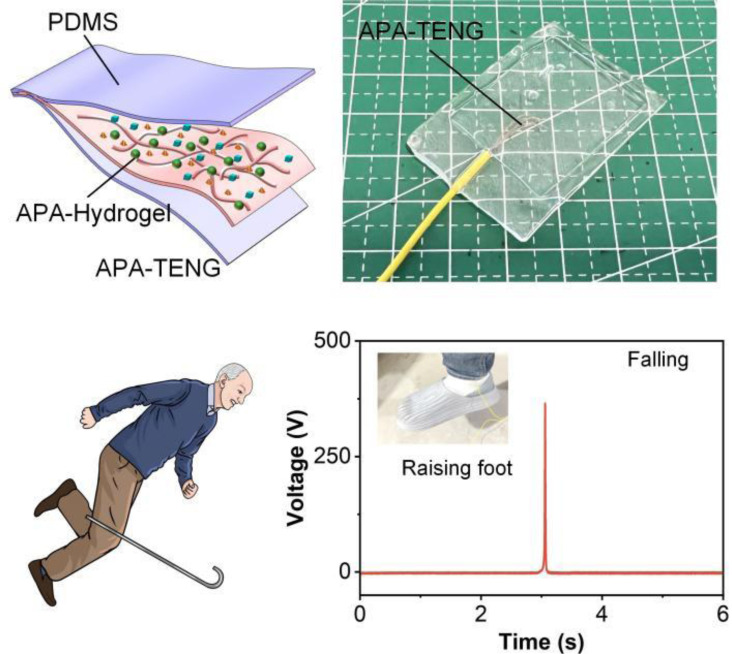

## 1 Introduction

With the rapid advancement of wearable electronic monitoring devices across various domains, particularly in fall detection,^[1]^ the Internet of Things (IoTs),^[2]^ wireless sensor networks,^[3]^ 3D printing,^[4]^ bio‐mechanical sensing,^[5]^ and human‐machine interfaces,^[6]^ the demand for power supply has become increasingly stringent. Modern wearable devices now require not only miniaturization, portability, and wearability, but also continuous monitoring and real‐time response capabilities.^[7]^ For instance, fall detection devices must continuously monitor human posture and issue timely alerts to prevent severe accidents.[^8^, ^9^] However, traditional power sources are constrained by limitations such as frequent charging requirements, short lifespan, large size, and rigid structure, making them inadequate for meeting the demands of next‐generation intelligent wearable devices. In this context, triboelectric nanogenerators (TENGs) have emerged as highly promising candidates for powering wearable electronics. TENGs offer several advantages, including ease of fabrication, low cost, lightweight construction, and environmental sustainability.[^10^, ^11^, ^12^, ^13^, ^14^, ^15^, ^16^, ^17^, ^18^, ^19^, ^20^] Moreover, they can effectively harvest mechanical energy from human motions such as walking,^[21]^ tapping,^[22]^ and joint bending,^[23]^ efficiently converting it into electrical energy to provide sustainable power for small wearable devices, including fall detection systems. Compared to conventional power technologies, TENGs significantly enhance the self‐powering capabilities of wearable electronics, enabling them to maintain reliable functional performance over extended periods and in complex environments. In the specific context of fall monitoring, powered triboelectric sensor devices are capable of delivering long‐term, stable energy supply, ensuring continuous and reliable real‐time monitoring while preventing power‐related interruptions.^[24]^ Additionally, the inherent flexibility of TENGs makes them particularly suitable for integration with wearable devices, as they can conform more comfortably to the contours of the human body compared to rigid power sources. This not only improves user comfort but also enhances the accuracy of data collection. As a result, TENG technology presents a new trajectory for the development of intelligent wearable systems, offering a robust technical foundation for applications in medical monitoring, health management, and beyond.^[25]^

Currently, TENGs operate in four distinct modes: vertical contact‐separation, lateral sliding, independent friction layer, and single‐electrode. Among these, single‐electrode TENGs have gained prominence in various application fields, particularly in wearable sensor systems, due to their simple structure, ease of use, and ability to directly adhere to human skin.^[26]^ The core components of a single‐electrode TENG are the friction layer and electrode layer, which together influence its electrical output performance.^[27]^ Key factors such as charge density, dielectric constant, material polarity, and surface area of the friction materials directly impact the generation of surface charges in the friction layer, as well as the charge transfer efficiency between different friction materials.[^28^, ^29^] Optimizing these parameters is essential for enhancing TENG's electrical output. Moreover, the mechanical properties of TENGs are critical, especially in wearable applications.^[30]^ The electrode materials must exhibit sufficient mechanical strength and flexibility under external forces to ensure the TENG device's stability and durability. Thus, electrode materials not only require high conductivity but also significant flexibility to meet the demands of bio‐mechanical sensing applications. Common electrode materials include metal plates,^[31]^ conductive polymers,^[32]^ and conductive hydrogels.^[33]^ Besides, artificial intelligence (AI) has emerged as a powerful tool for accelerating the discovery and optimization of novel materials and devices.^[34]^ By leveraging AI‐based computational methodologies, researchers can predict material properties, optimize device performance, and design next‐generation technologies with enhanced efficiency and precision.^[35]^ For instance, AI‐driven approaches have been applied in energy storage systems, super‐capacitor materials, and sustainable energy devices.[^36^, ^37^] Metal and carbon plates, although highly conductive, lack stretchability, which restricts their suitability for flexible wearable devices.^[38]^ Conductive polymers offer greater flexibility but are often limited by complex fabrication processes. In contrast, conductive hydrogels are regarded as ideal materials for wearable applications due to their superior flexibility and extensibility.^[39]^ However, in practical applications, the adhesion between electrode materials and triboelectric layers is a critical factor, as poor adhesion can significantly degrade device performance. Therefore, when designing TENGs for wearable sensors, it is essential to consider not only electrical and mechanical properties but also the adhesion of the electrode to ensure long‐term operational stability.[^40^, ^41^] Despite significant progress in TENG technologies, many conventional designs face challenges such as limited energy output, lack of flexibility, and reduced durability under repeated mechanical deformation. Existing conductive hydrogel‐based TENGs often suffer from poor ionic conductivity or mechanical instability, which limits their applicability in wearable and assistive devices.

In this study, a novel double‐network (DN) conductive hydrogel, designated as Agarose/P(HEA‐co‐AA)‐Al/NaCl (APA‐hydrogel), was successfully synthesized. This hydrogel comprises two interlinked networks: the first, formed by agarose–a biomacromolecule rich in hydroxyl (−OH) groups–provides a stable structural framework. The second network is constructed from poly (2‐hydroxyethyl acrylate‐co‐acrylic acid) (P(HEA‐co‐AA)), which is reinforced through the coordination of aluminum ions (Al^3+^) with carboxylate (COO^−^) groups. To enhance its ionic conductivity, the APA‐hydrogel was immersed in a NaCl solution. The sodium ions (Na^+^) in the solution attracted water molecules, resulting in the formation of hydrated sodium ions ([Na(H_2_O)_n_]^+^), which were anchored within the hydrogel matrix via hydrogen bonding and metal coordination. This process not only stabilized the hydrated ions within the network but also contributed significantly to the hydrogel's enhanced mechanical and electrical properties. The resulting APA‐hydrogel was then utilized as the core material for a triboelectric nanogenerator (APA‐TENG). By serving as a conductive electrode, the APA‐hydrogel enabled efficient energy harvesting, showcasing its potential for advanced applications in wearable and assistive technologies. The proposed APA‐TENG operates in the contact‐separation mode, which is particularly suitable for applications requiring efficient energy harvesting from periodic motions. This mode ensures effective charge transfer between the triboelectric layers during the contact and separation process, contributing to the high electrical output and stability observed in the APA‐TENG. According to the experimental data, the SP‐TENG (with a surface area of 8 cm^2^) achieved an open‐circuit voltage (V_oc_) of 900 V, a short‐circuit current (I_sc_) of 73.42 μA, and a transferred charge (Q_sc_) of 229 nC. The peak power output was recorded at 3 MΩ load resistance, where the APA‐TENG device produced around 3.52 mW of power. The APA‐TENG demonstrates significant potential for various practical applications, including powering low‐power electronic devices and assistive technologies. It efficiently harvests mechanical energy, stores it, and powers devices like LEDs, while also functioning as a real‐time sensor for motion and fall detection, making it suitable for wearable and assistive systems that capitalize on human activities for energy generation.

## Experiments

### Materials

Hydroxyethyl acrylate (HEA) powders were obtained from Shandong Huaye Trading Co., Ltd., Ltd, China. Acrylic acid (AA) powders were acquired from Jinan Chenwang Chemical Technology Co., Ltd, China. Industrial aluminium chloride (AlCl_3_) and sodium chloride (NaCl) powders were bought from Jinan Duanxing Chemical Technology Co., Ltd, China. Polydimethylsiloxane (PDMS) (Sylgard™ 184) was purchased from Guangzhou Yinuo Chemical Technology Co., Ltd, China. The agarose was bought from Shenzhen Doudian Biotechnology Co., Ltd, China. Ammonium persulfate (APS) was obtained from Shanghai Senxingyan Biotechnology Co., Ltd, China. In addition, industrial‐grade deionized water was purchased from Jinan Gongchuang Biotechnology Co., Ltd, China, while wires, culture dishes, beakers, and other tools were sourced from the mall.

### The Preparation Process of APA‐Hydrogel and APA‐TENG Device

**Figure 1:**
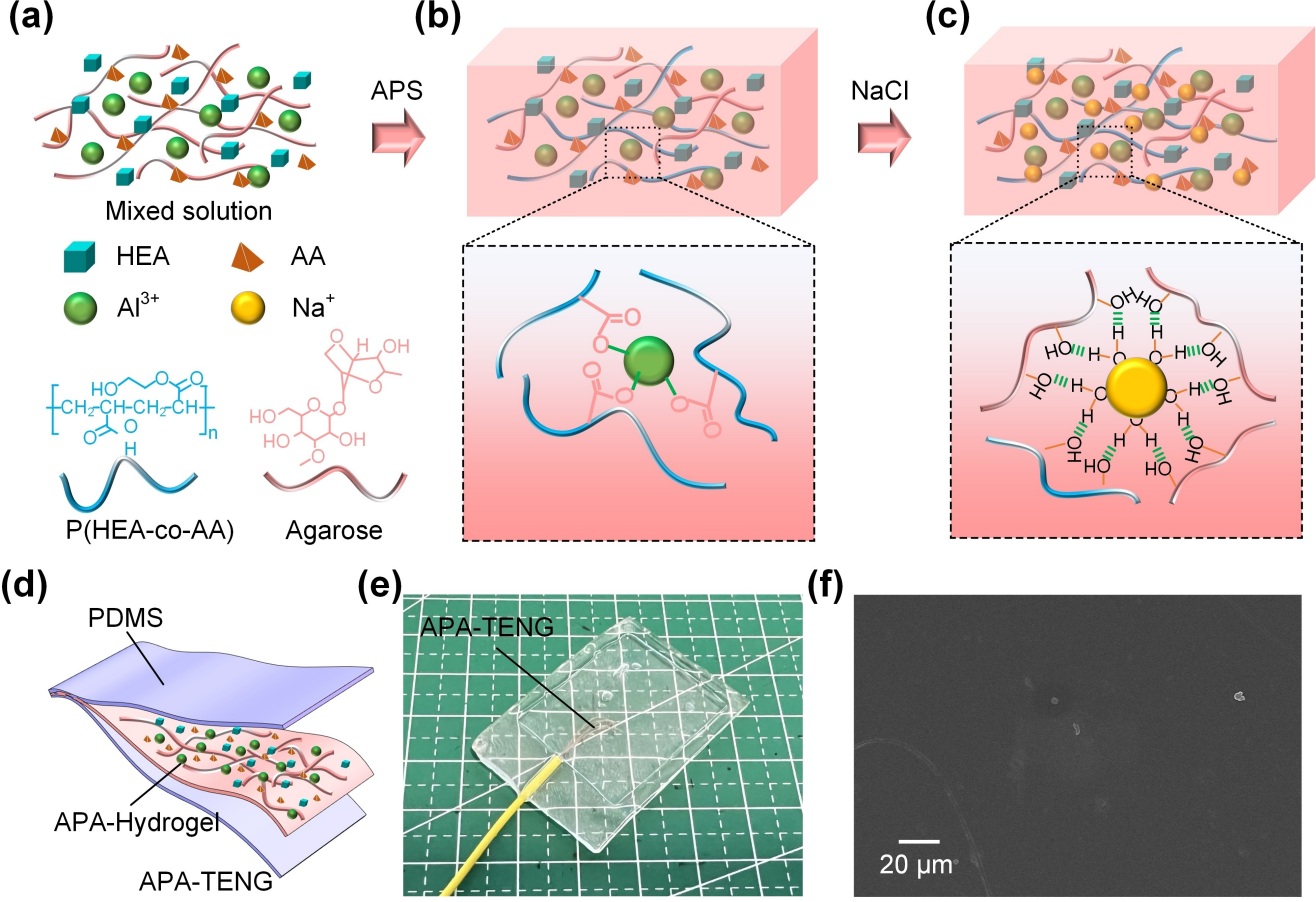
Schematic and structural representation of the APA‐TENG device. (a) Mixed solution containing HEA, AA, Al^3+^, Na^+^, and agarose. (b) Polymerization process initiated by APS, forming a hydrogel network via Al^3+^ coordination with carboxyl groups. (c) NaCl treatment introduces Na^+^, creating hydration shells and hydrogen bonds to enhance the hydrogel structure. (d) Structure of the APA‐TENG with APA‐hydrogel sandwiched between PDMS layers. (e) Photograph of the fabricated APA‐TENG. (f) The SEM image showing the hydrogel microstructure.

In Figure 1(a), a mixed solution containing hydroxyethyl acrylate (HEA), acrylic acid (AA), aluminum ions (Al^3+^), and sodium ions (Na^+^) is shown. The molecular structures of the copolymer P(HEA‐co‐AA) and agarose are depicted, emphasizing the role of the Al^3+^ ion in coordinating with the carboxyl groups in the polymer matrix. This coordination enhances the structural stability of the APA‐hydrogel. Figure 1(b) demonstrates the polymerization process initiated by ammonium persulfate (APS), where the HEA and AA monomers copolymerize, forming a robust network through the coordination of Al^3+^ with carboxylate (COO^−^) groups. This step results in the formation of a hydrogel structure with Al^3+^ ions embedded in the polymer network, creating a firm and cohesive structure. In Figure 1(c), the APA‐hydrogel is subjected to NaCl treatment. This process allows Na^+^ ions to diffuse into the hydrogel network, where they interact with hydroxyl (−OH) and carboxyl groups through hydrogen bonding, further enhancing the mechanical properties and the ionic conductivity of the hydrogel. The illustration highlights how the Na^+^ ions form hydration shells, establishing multiple hydrogen bonds within the network. Then, the prepared PDMS film is wrapped on the surface of APA‐hydrogel, and the interface is sealed with PDMS solution. Furthermore, the device is dried to solidify the PDMS solution. Figure 1(d) shows the assembly of the APA‐TENG device, where the APA‐hydrogel is sandwiched between two PDMS layers, forming the complete TENG device.

### Characterization and Measurements

Figure 1(e) presents an actual photograph of the fabricated APA‐TENG device on a grid mat, while Figure 1 (f) provides a scanning electron microscope (SEM) image of the APA‐hydrogel structure, revealing the fine details of its microstructure. The V_oc_ of the APA‐TENG was measured with the help of a digital oscilloscope (DS1102Z‐E) to capture precise voltage output data. Simultaneously, the I_sc_ and Q_sc_ were carefully recorded using a Keithley 6514 electrometer, ensuring accurate measurement of electrical parameters. To simulate the required mechanical forces for actuation, a custom‐designed adjustable mechanical motor was employed, providing controlled and repeatable force application. This setup allowed for detailed evaluation of the APA‐TENG's electrical performance under different mechanical conditions.

## 2 Results and Discussion

**Figure 2:**
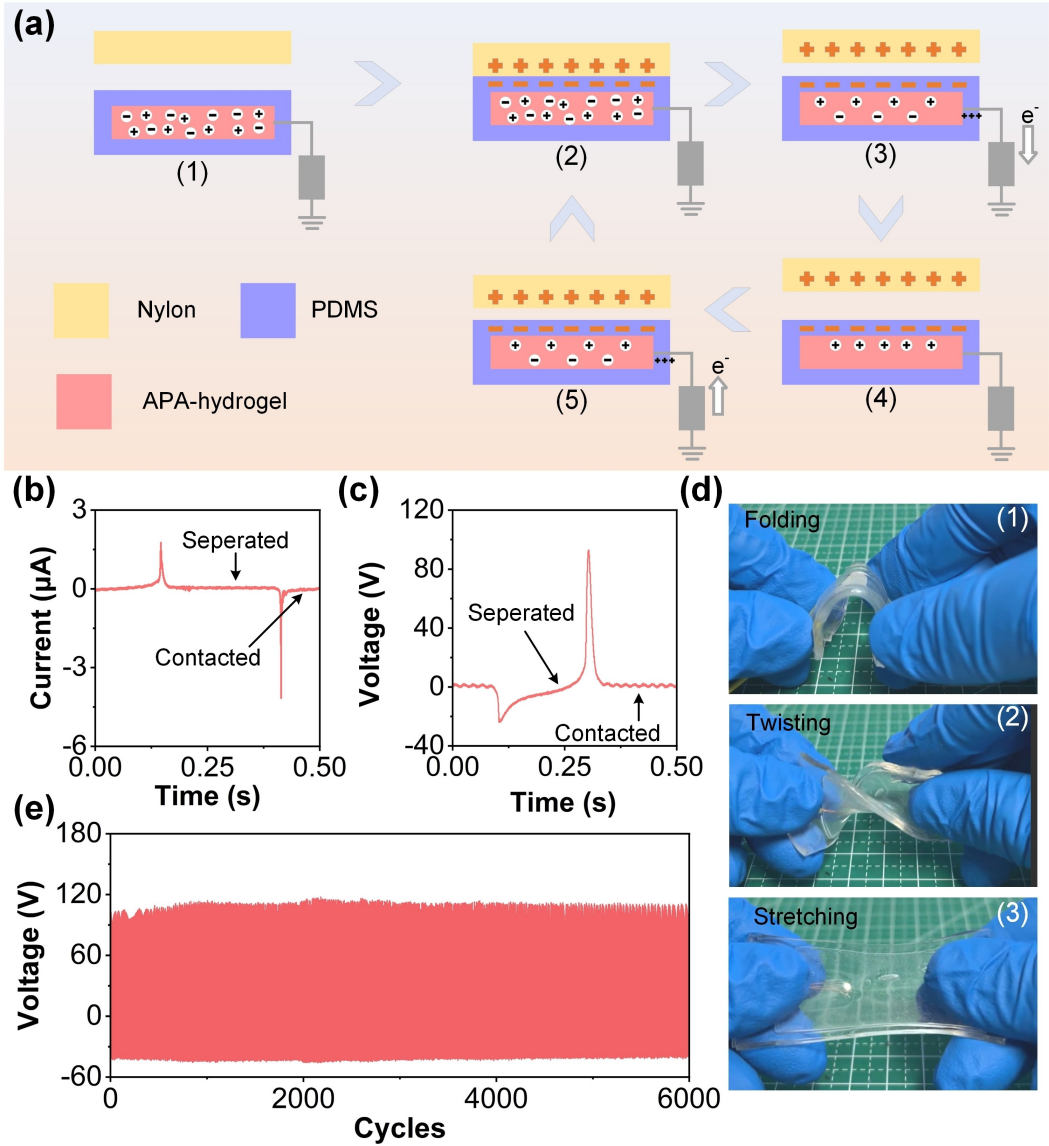
Working principle, operational stability, and structural flexibility of the APA‐TENG. (a1–a5) Schematic diagram of the working mechanism of the TENG, illustrating the contact and separation processes between Nylon, PDMS, and APA‐hydrogel layers. (b) Current output during the contact‐separation cycle. (c) Voltage output during the contact‐separation cycle. (d1–d3) Mechanical deformation tests of the TENG under folding, twisting, and stretching. (e) Long‐term stability of the TENG with voltage output maintained over 6000 cycles.

The ionic conductivity of the APA‐hydrogel is influenced by the concentration of NaCl, as the sodium ions (Na^+^) increase the availability of free charge carriers within the hydrogel matrix. At lower NaCl concentrations, the conductivity rises rapidly due to the increased number of dissociated ions. However, as the concentration continues to increase, the conductivity eventually plateaus because of the saturation of ionic mobility within the double‐network structure. This relationship between NaCl concentration and ionic conductivity contributes significantly to the enhanced electrical output of the APA‐TENG, as evidenced by the trends observed in the open‐circuit voltage and short‐circuit current. Although the APA‐hydrogel serves as the electrode rather than the frictional layer, the NaCl concentration significantly influences the TENG′s performance by enhancing the ionic conductivity of the hydrogel. As the NaCl concentration increases, more free ions are available within the hydrogel matrix, which facilitates efficient charge transport and reduces internal resistance. This improved conductivity ensures more effective charge transfer from the electrode to the external circuit, maximizing the electrical output of the TENG. The enhanced interaction between the highly conductive hydrogel and the triboelectric charges generated at the PDMS‐Nylon interface underscores the critical role of the NaCl‐coordinated hydrogel in optimizing device performance. Figure 2(a) depicts a five‐stage cycle demonstrating the working principle of the TENG, with the contact‐separation mode between the triboelectric layers (PDSM layer@Nylon). In Figure 2(a1), the nylon layer and the PDMS layer are in an initial uncontacted state. Figure 2(a2) shows the initiation of contact between the nylon and APA‐hydrogel through an intermediary PDMS layer, which is responsible for charge obtaining. The positive and negative charges start accumulating on the respective surfaces due to triboelectric effects when the materials are in contact. Figure 2(a3) indicates the charge transfer as the materials are separated, and electrons flow toward the external circuit, represented by the e^−^ symbol. Figure 2(a4) illustrates the reverse flow of electrons when the materials come back into contact, and finally, Figure 2(a5) completes the cycle with charges returning to the neutral state. This cyclical process is critical for generating electrical energy through the repetitive contact and separation of the layers. The current output of the APA‐TENG is shown in Figure 2(b), which plots current (μA) versus time (s). A peak current of approximately 3 μA is observed during the separation phase, which is labeled in the figure. This peak corresponds to the generation of electrical current when the materials are separated, while the dip in the current when the materials come into contact indicates the flow of electrons back into the system, leading to current reduction. The voltage output is displayed in Figure 2(c), where voltage (V) is plotted against time (s). The voltage exhibits a peak around 100 V during the separation phase, which correlates with the maximum potential difference generated when the triboelectric layers are separated. Similar to the current profile, the voltage decreases during the contact phase as charges redistribute. This demonstrates the APA‐TENG′s effective charge generation and electrical performance in the contact‐separation mode. Figure 2(d) shows the APA‐TENG under different mechanical deformations, including folding, twisting, and stretching. Each subfigure (1–3) highlights the APA‐TENG's mechanical resilience and flexibility, essential for practical applications in flexible electronics. Video 1 shows various deformation states of APA‐hydrogel in detail. The APA‐TENG maintains its structural integrity under all three deformation modes, indicating the robustness of the APA‐hydrogel layer and its compatibility with flexible substrates. Figure 2(e) represents the voltage output over a prolonged testing period. The voltage remains stable around 100 V throughout 6000 cycles, confirming the APA‐TENG′s durability and consistent performance over extended operational periods. Figure S1(a) of Supporting Information shows the schematic representation of the device, where the APA‐hydrogel serves as the triboelectric positive layer, interacting with a negative triboelectric material under periodic contact‐separation motions. The electrical output performance is evaluated in terms of voltage, current, and charge. Figure S1(b) of Supporting Information displays the voltage output of the system, with a peak value of approximately 46.63 V, which is significantly lower compared to conventional triboelectric materials. Figure S1(c) of Supporting Information presents the current output, with a maximum peak value of 4.91 μA. Similarly, Figure S1(d) of Supporting Information depicts the charge transfer performance, with a peak charge of 14 nC. These results highlight the relatively low output performance of the APA‐hydrogel as a triboelectric material, attributed to its limited ability to generate and sustain high triboelectric charges. While APA‐hydrogel offers advantages such as flexibility and biocompatibility, the reduced electrical output limits its applications in scenarios requiring high‐performance energy harvesting. These findings suggest that material modifications or hybrid designs may be necessary to enhance the triboelectric output when utilizing hydrogel‐based triboelectric materials. Figure S2 of Supporting Information shows TENG test scenarios under different forces.

**Figure 3:**
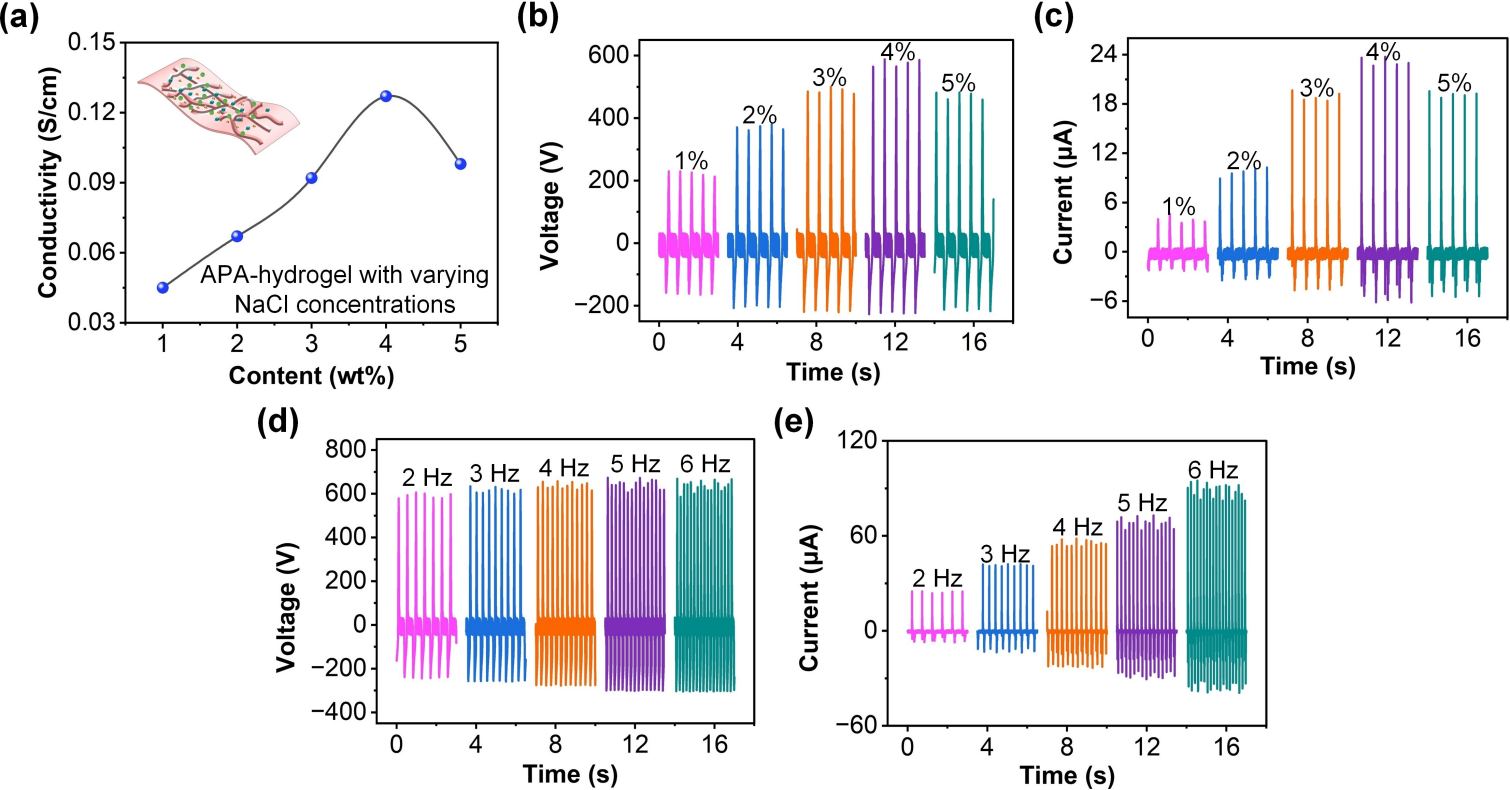
Electrical performance of the APA‐TENG under different NaCl concentrations and working frequencies. (a) The electrical conductivity of APA hydrogel with varying NaCl concentrations. The effects of NaCl concentration (1 %, 2 %, 3 %, 4 %, and 5 %) on the (b) V_oc_ and (c) I_sc_.The effects of working frequency (2 Hz, 3 Hz, 4 Hz, 5 Hz, and 6 Hz) on the (d) V_oc_ and (e) I_sc_.

Figure 3(a) shows the relationship between the electrical conductivity of APA hydrogel and varying NaCl concentrations. The curve indicates that as the NaCl concentration increases, the conductivity of the hydrogel first rises, reaching a peak before decreasing. The conductivity ranges from approximately 0.04 S/cm to 0.12 S/cm, highlighting the significant impact of NaCl concentration on the APA hydrogel′s electrical properties. In Figure 3(b), the V_oc_ progressively increases as the NaCl concentration rises, from around 200 V at 1 % concentration to nearly 600 V at 5 %. This indicates that higher ion concentration within the APA‐hydrogel enhances the triboelectric performance due to increased ion mobility and surface charge density. The I_sc_ output follows a similar trend, with a maximum current of approximately 24 μA at 5 % NaCl concentration, demonstrating a direct relationship between ionic content and current generation capacity, as illustrated in Figure 3(c). The generated Q_sc_ also increases with NaCl content, reaching approximately 180 nC at 5 %, suggesting that the presence of NaCl significantly influences the amount of charge separation and overall energy output of the TENG, as shown in Figure S3 of Supporting Information. These results demonstrate that higher NaCl concentrations lead to enhanced charge transfer and thus higher electrical output, making ion concentration a critical factor in optimizing the APA‐TENG′s performance. In Figure 3(d), the V_oc_ increases slowly with frequency, reaching about 700 V at 6 Hz. The consistent rise in voltage suggests that the faster contact‐separation cycles promote greater charge accumulation and discharge, enhancing the energy output. Similar to the voltage, the I_sc_ output in Figure 3(e) also increases with frequency, with a maximum value of around 110 μA at 6 Hz. This indicates a strong correlation between the frequency of mechanical motion and current generation. In Figure S4 of Supporting Information, the generated Q_sc_ increases slowly with frequency, reaching nearly 200 nC at 6 Hz. This trend reinforces the relationship between working frequency and charge accumulation, showing that higher operational speeds result in more efficient charge generation. In summary, both NaCl concentration and working frequency have a significant impact on the electrical performance of the APA‐TENG. Higher NaCl content leads to enhanced ion mobility and surface charge density, while higher frequencies promote faster and more efficient charge separation, collectively optimizing the APA‐TENG's output for energy harvesting applications.

**Figure 4:**
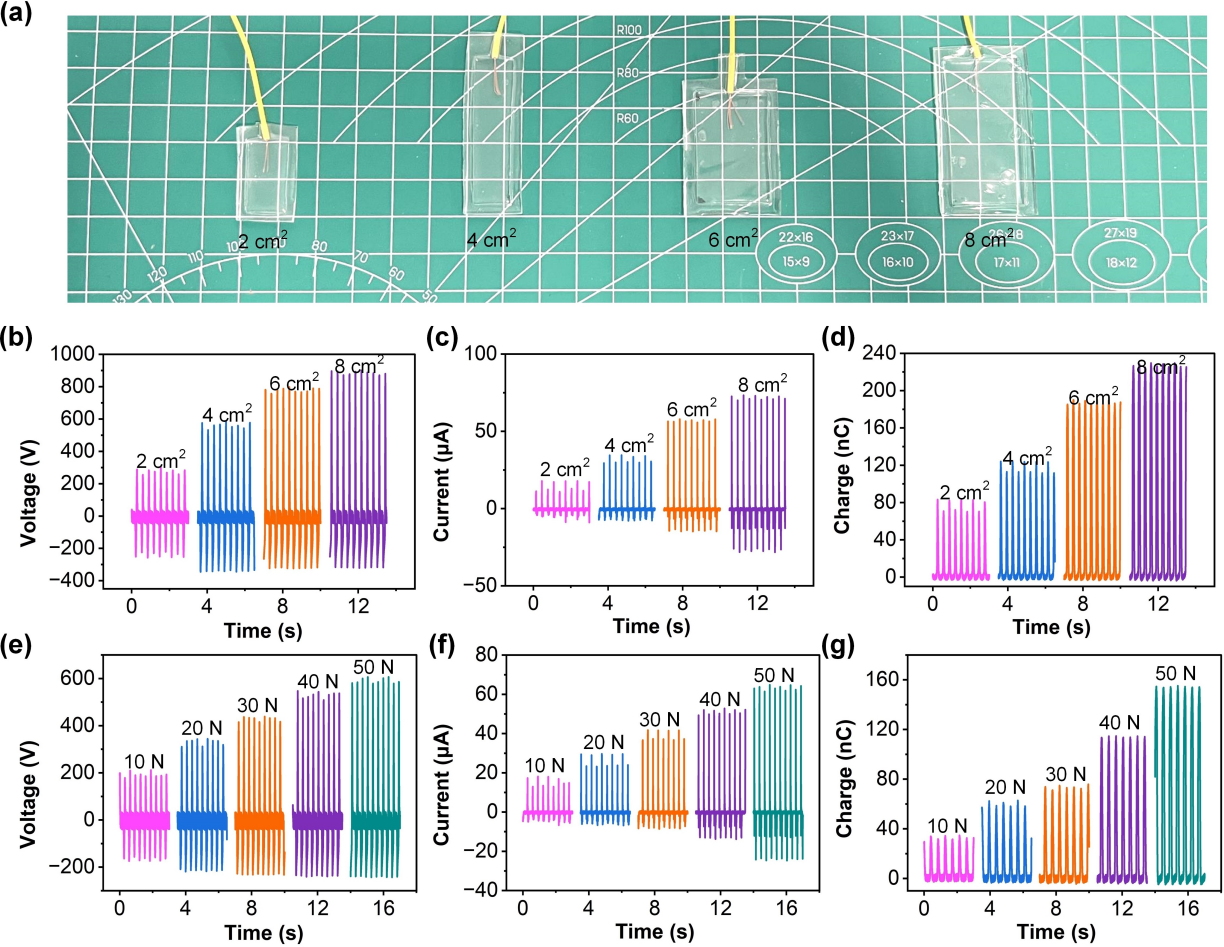
Electrical performance of APA‐TENG under varying surface areas and applied pressures. (a) The pictures of APA‐TENG in different sizes. The effects of surface area (2 cm^2^, 4 cm^2^, 6 cm^2^, and 8 cm^2^) on the output (b) V_oc_, (c) I_sc_, and (d) Q_sc_. The effects of applied pressure (10 N, 20 N, 30 N, 40 N, and 50 N) on the output (e) V_oc_, (f) I_sc_, and (g) Q_sc_.

Figure 4(a) illustrates the physical design and variations in the contact area of the APA‐TENG used for the experimental analysis. The images show four distinct configurations of the device with contact areas of 2 cm^2^, 4 cm^2^, 6 cm^2^, and 8 cm^2^, respectively. The devices are placed on a measurement grid for clarity and size comparison. These variations in contact area are crucial for understanding the relationship between the effective triboelectric area and the output performance of the APA‐TENG, including voltage, current, and charge generation. By systematically increasing the contact area, the experiments aim to evaluate how the surface interaction impacts the energy harvesting capabilities of the device, providing valuable insights into optimizing the device for enhanced performance. Figure 4(b–d) show the V_oc_, I_sc_, and Q_sc_ outputs, respectively, with different active surface areas of the APA‐TENG: 2 cm^2^, 4 cm^2^, 6 cm^2^, and 8 cm^2^. In Figure 4(b), the V_oc_ output increases as the surface area expands, from around 302 V at 2 cm^2^ to nearly 900 V at 8 cm^2^. This trend indicates that a larger surface area results in a higher amount of charge generation due to the increased contact area between the triboelectric layers. In Figure 4(c), the I_sc_ output shows a similar pattern, with values rising from approximately 17.96 μA at 2 cm^2^ to nearly 73.42 μA at 8 cm^2^. This increase is attributed to the enhanced charge density as the surface area grows. The Q_sc_ output also rises with the surface area, reaching around 229 nC at 8 cm^2^, as shown in Figure 4(d). The linear increase in charge with the contact area demonstrates that the larger the active area of the APA‐TENG, the more effective it is at generating triboelectric charges, resulting in higher electrical output. Figure 4(e–g) show the output performance of the APA‐TENG under different applied pressures: 10 N, 20 N, 30 N, 40 N, and 50 N. In Figure 4(e), the V_oc_ output increases steadily with applied pressure, reaching a maximum of around 606 V at 50 N. Higher pressure enhances the contact intimacy between the triboelectric layers, leading to more efficient charge transfer. Similarly, the I_sc_ output increases with applied pressure in Figure 4(f), reaching nearly 64.88 μA at 50 N. This trend further confirms the role of pressure in facilitating better contact between the surfaces, which in turn boosts the current generation. In Figure 4(g), the Q_sc_ output also increases with applied pressure, reaching approximately 155 nC at 50 N. The enhancement in charge output with greater pressure highlights the pressure sensitivity of the APA‐TENG, making it suitable for applications where varying forces are involved. Hence, both surface area and applied pressure are crucial factors influencing the electrical output of the APA‐TENG. Larger surface areas and higher applied pressures significantly enhance voltage, current, and charge outputs, providing a clear path for optimizing the device‘s performance in energy harvesting applications. Figure S5(a) of Supporting Information presents the voltage output as a function of time at different humidity levels. At 20 % relative humidity, the device exhibits a peak output voltage of approximately 358 V, which decreases steadily with increasing humidity, showing a reduced peak voltage at 80 %. This trend highlights the influence of moisture on the triboelectric effect, where the presence of water molecules diminishes charge transfer efficiency. Figure S5(b) of Supporting Information illustrates the current output under the same conditions. Similar to the voltage output, the peak current decreases as the humidity increases, ranging from approximately 26.55 μA at 20 % to significantly lower values at 80 %. This decline is consistent with the reduced triboelectric performance in more humid environments. Figure S5(c) of Supporting Information displays the charge output behavior over time. The device generates a peak charge of around 59 nC at 20 % humidity, with a gradual decline observed at higher humidity levels. This trend further confirms the detrimental effect of water adsorption on the triboelectric charge generation and retention. These results collectively demonstrate the sensitivity of the APA‐TENG′s performance to environmental humidity, which must be considered when optimizing its application in real‐world scenarios. Adjustments in material selection or device encapsulation may be required to mitigate performance degradation in high‐humidity conditions.

**Figure 5:**
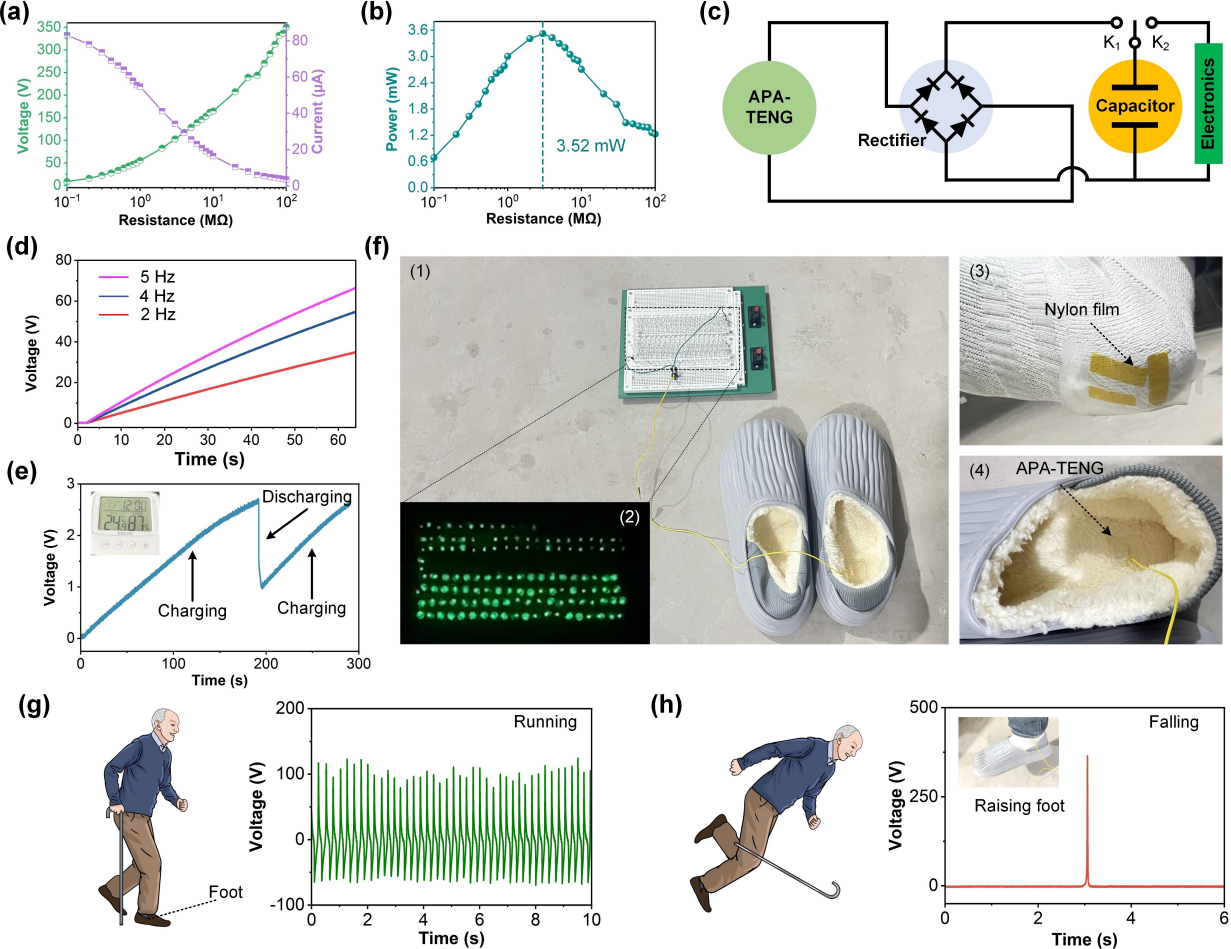
Electrical performance and applications of the APA‐TENG. (a) Voltage and current output as a function of resistance. (b) Power output with peak value of 3.52 mW at optimal resistance. (c) Circuit design for energy storage using a rectifier and capacitor. (d) Voltage output at different frequencies (2 Hz, 4 Hz, and 5 Hz). (e) Charging and discharging process of stored energy. (f1) Experimental setup with APA‐TENG embedded in shoes and connected to a circuit. (f2) LED array powered by APA‐TENG during walking. (f3) Nylon film on the insole for enhanced triboelectric interaction. (f4) APA‐TENG embedded in the insole, highlighting its compact and ergonomic design. (g) Voltage output during running motion with APA‐TENG integrated into an assistive device. (h) Fall detection indicated by a sharp voltage spike.

The output voltage and current as functions of external load resistance for the APA‐TENG under controlled mechanical testing conditions. These values represent a specific test configuration and differ from the maximum output metrics reported under optimized conditions. Figure 5(a) presents the variation of output voltage and current as a function of external load resistance. The green curve shows the voltage increasing with increasing resistance, peaking around 300 V at approximately 10 MΩ. In contrast, the current (purple curve) decreases with increasing resistance, reaching values near zero at high resistances. This inverse relationship between voltage and current is characteristic of energy‐generating devices, indicating optimal load matching. Figure 5(b) shows the power output as a function of resistance, peaking at 3.52 mW at a load resistance of around 10 MΩ. This indicates that the APA‐TENG (size: 8 cm^2^) achieves its highest power generation efficiency at this specific load, making it suitable for driving low‐power electronics under optimized conditions. The calculated power density is 44 mW/m^2^, which aligns well with the performance of high‐output TENGs. Figure 5(c) illustrates the circuit design employed for energy harvesting, storage, and utilization. The APA‐TENG is connected to a full‐wave bridge rectifier, which converts the alternating output of the APA‐TENG into direct current, followed by a capacitor for energy storage. Two switches (K1 and K2) control the charging and discharging processes, allowing for efficient power management in practical applications. Figure 5(d) displays the voltage output of the APA‐TENG at different operating frequencies (2 Hz, 4 Hz, and 5 Hz). The voltage output increases linearly with time and at higher frequencies, with 5 Hz generating the highest voltage of approximately 70 V after 60 s. This demonstrates the frequency sensitivity of the APA‐TENG, where faster contact‐separation cycles enhance energy generation. Figure 5(e) presents the voltage profile of the APA‐TENG during a charging and discharging process, where the voltage steadily rises during the charging period and drops abruptly during discharge, as indicated by the sharp voltage drop. This highlights the APA‐TENG's capability for energy storage and its potential use in applications requiring intermittent energy supply. Figure 5(f1) shows the overall setup, where the APA‐TENG (size: 2 cm×2 cm), embedded in the shoe‘s insole and equipped with a nylon film for enhanced triboelectric interactions, is connected to an external circuit board to harvest bio‐mechanical energy. Figure 5(f2) highlights the device‘s ability to power 140 light emitting diodes (LEDs), with the illuminated LEDs demonstrating the efficient conversion of bio‐mechanical energy into electrical energy in real time. The Video 2 shows the use of foot movement to drive LEDs. Figure 5(f3) provides a close‐up of the APA‐TENG′s structural integration, emphasizing the strategic placement of the nylon film within the shoe to optimize energy harvesting during foot motion. Finally, Figure 5(f4) offers a detailed view of the APA‐TENG embedded within the insole, showcasing its compact design, secure assembly, and ergonomic integration to ensure both energy harvesting efficiency and user comfort. This comprehensive setup illustrates the feasibility of APA‐TENG‐based wearable devices for sustainable energy harvesting applications. Figure 5(g) showcases the voltage output of the APA‐TENG when integrated into an assistive device during running. The schematic on the left illustrates a user running with a cane equipped with an APA‐TENG, while the graph on the right shows the corresponding voltage profile. The regular voltage peaks suggest consistent energy generation during motion, demonstrating the device‘s potential for energy harvesting from human activities. Figure 5(h) shows the APA‐TENG′s application in fall detection. The left panel depicts a schematic of a user falling, while the right panel displays the voltage output during this event. A sharp voltage spike is observed upon the fall, indicating that the APA‐TENG can serve as a real‐time fall detection sensor based on sudden motion changes, which generate significant voltage deviations. Hence, the versatility and efficiency of the APA‐TENG in various energy harvesting and sensing applications. The data emphasize its ability to operate under different resistive loads and frequencies, store energy, power electronic devices, and serve as a motion and fall detection sensor, making it highly suitable for assistive technologies.

## 3 Conclusions

In conclusion, a novel double‐network (DN) conductive APA‐hydrogel was successfully synthesized. The first network, formed by agarose, provides a stable framework, while the second network, constructed from P(HEA‐co‐AA) via aluminum ion coordination, enhances structural integrity. After immersion in NaCl solution, sodium hydrate ions formed and stabilized within the hydrogel matrix. The resulting APA‐hydrogel was applied in APA‐TENG, demonstrating impressive performance with an V_oc_ of 900 V, a I_sc_ of 73.42 μA, and a Q_sc_ of 229 nC. With a peak power output of 3.52 mW at 3 MΩ load resistance, the APA‐TENG shows strong potential for powering low‐power devices and assistive technologies. It efficiently harvests mechanical energy and functions as a real‐time sensor for applications such as motion and fall detection, making it ideal for wearable systems powered by human activity.

## Conflict of Interests

The authors declare no conflict of interest.

## Supporting information

As a service to our authors and readers, this journal provides supporting information supplied by the authors. Such materials are peer reviewed and may be re‐organized for online delivery, but are not copy‐edited or typeset. Technical support issues arising from supporting information (other than missing files) should be addressed to the authors.

Supporting Information

Supporting Information

Supporting Information

## Data Availability

The data that support the findings of this study are available from the corresponding author upon reasonable request.
