# Supplementary material for: A Flexible and Stretchable Triboelectric Nanogenerator with Agarose/P(HEA‐co‐AA)‐Al/NaCl Electrodes for Bio‐Mechanical Energy Harvesting and Fall Detection
Source: ChemistryOpen. 2025 Mar 5;14(6):e202400394. doi: 10.1002/open.202400394 (PMC12138051; doi:10.1002/open.202400394)
Supplement: Supplementary file 1 — Supporting Information [file OPEN-14-e202400394-s002.pdf]

ChemistryOpen

Supporting Information

A Flexible and Stretchable Triboelectric Nanogenerator with Agarose/P(HEA-co-AA)-Al/NaCl Electrodes for Bio-Mechanical Energy Harvesting and Fall Detection

Xiwei Liu and Hui Zhang*

A Flexible and Stretchable Triboelectric Nanogenerator with Agarose/P(HEA-co-AA)-Al/NaCl Electrodes for Bio-mechanical Energy Harvesting and Fall Detection

Xiwei Liu[1], Hui Zhang[2*]

[1]Sports College Lanzhou City University, Lanzhou City, Gansu Province, 730070, China;
[2]Military Sports Department Changchun Sci-Tech University, Changchun, 130600, China.

*Corresponding author: 100481@cstu.edu.cn.

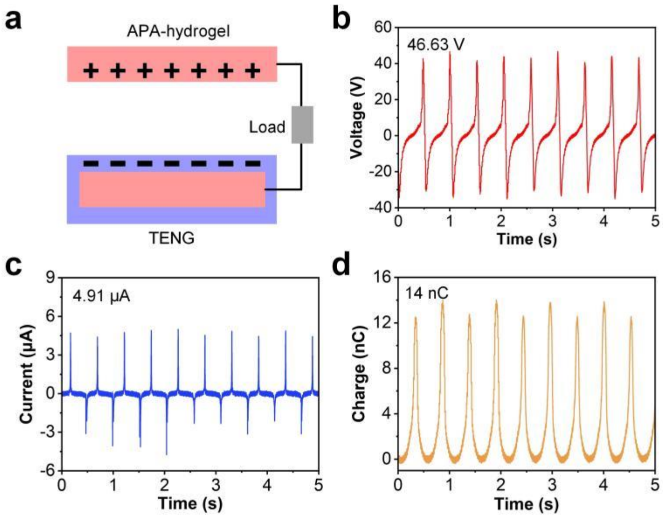

Fig. S1. (a) The structural diagram of APA-TENG using APA-hydrogel as the triboelectric layer. The (b) V_{oc}, (c) I_{sc}, (d) Q_{sc} of APA-TENG using APA-hydrogel as the triboelectric layer.

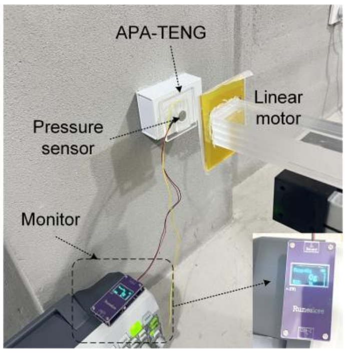

Fig. S2. The APA-TENG test scene images under different forces.

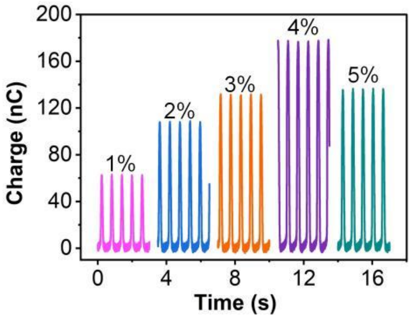

Fig. S3. The effects of NaCl concentration (1%, 2%, 3%, 4%, and 5%) on the Q_{sc} of APA-TENG.

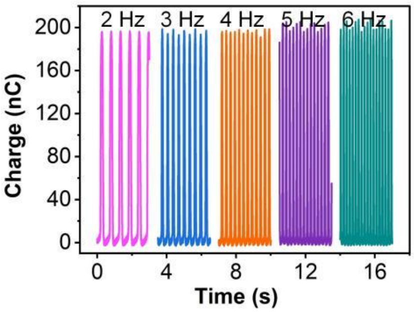

Fig. S4. The effects of working frequency (2 Hz, 3 Hz, 4 Hz, 5 Hz, and 6 Hz) on the Q_{sc} of APA-TENG.

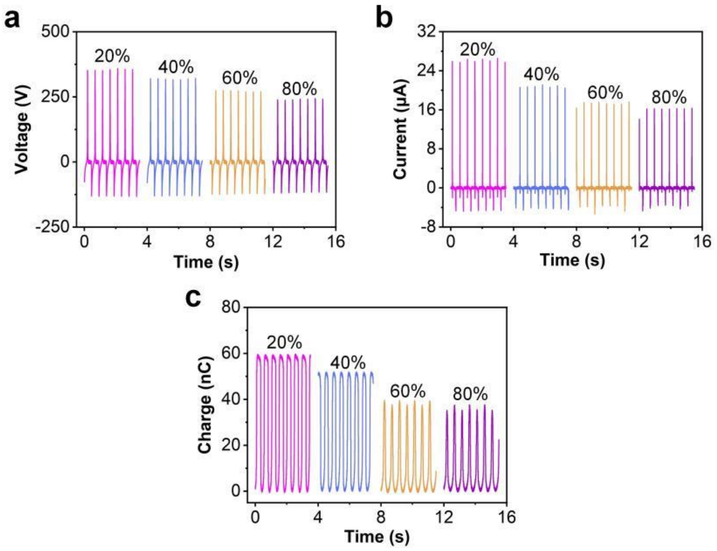

Fig. S5. The (a) V_{oc}, (b) I_{sc}, and (c) Q_{sc} of APA-TENG under different humidity conditions.
